# Characterization of starch extracted from seeds of *Cycas revoluta*

**DOI:** 10.3389/fnut.2023.1159554

**Published:** 2023-05-25

**Authors:** Kehu Li, Tongze Zhang, Wei Zhao, Huanhuan Ren, Siqi Hong, Yongyi Ge, Harold Corke

**Affiliations:** ^1^Key Laboratory of Plant Resource Conservation and Germplasm Innovation in Mountainous Region (Ministry of Education), Collaborative Innovation Center for Mountain Ecology & Agro-Bioengineering (CICMEAB), Institute of Agro-Bioengineering, College of Life Sciences, Guizhou University, Guiyang, Guizhou, China; ^2^Biotechnology and Food Engineering Program, Guangdong Technion-Israel Institute of Technology, Shantou, China; ^3^Faculty of Biotechnology and Food Engineering, Technion-Israel Institute of Technology, Haifa, Israel

**Keywords:** *Cycad revoluta*, starch, physicochemical properties, amylopectin structure, food bioresources

## Abstract

**Introduction:**

Starch is major component in the big seeds of *Cycas revoluta*, however the characteristics of *Cycas revoluta* remain unknown.

**Methods:**

In this study, the physicochemical and structural properties of two starch samples extracted from *Cycad revoluta* seeds were systematically investigated, using various techniques.

**Results:**

The amylose contents of the two samples were 34.3 % and 35.5%, respectively. The spherical-truncated shaped starch granules possessed A-type crystallinity, and had an average diameter less than 15 μm. Compared to most commonly consumed cereal and potato starch, *Cycad revoluta* starch showed distinctive characteristics. For physicochemical properties, in the process of gelatinization, the *Cycad revoluta* starch showed similar viscosity profile to starches of some potato varieties, but *Cycad revoluta* starch had higher gelatinization temperature. Upon cooling, *Cycad revoluta* starch formed harder gels than rice starch. For structure, the molecular weight (indexed by Mw, Mn and Rz values), branching degree and the branch chain length distribution were determined.

**Discussion:**

The results suggested that *Cycad revoluta* starch were different in structure from the main-stream starches. Notable differences in some starch traits between the two samples were recorded, which could be attributed to environmental factors. In general, this study provides useful information on the utilization of *Cycad revoluta* starch in both food and non-food industries.

## 1. Introduction

*Cycas* plants belong to the family Cycadaceae, which consists of 11 genera of plants. Among them, *Cycas revoluta*, the “Sago palm,” is the most cultivated species and mainly planted for uses in ornamental horticulture (1). *Cycas revoluta* produces terminal oblong cones containing orange-yellow big seeds, which are potential of nutritional significance for humans. However, study on the nutritional components in seeds of *Cycas revoluta* is very limited. Starch is usually the major nutritional component in plant seeds, characterization of the seed starch of *Cycas revoluta* is of importance, as we might be able to find novel starch with unique quality that could be used in food and non-food industry.

Starch is mainly composed of two types of polysaccharides: amylose with few branches and highly branched amylopectin. Starch quality is determined by its characteristics including amylose content, fine structure of amylopectin, and the shape and size of starch granules (2, 3). These characteristics also determine starch utilization, for example, starch with low amylose content is preferred in making fermented cake, while those with intermediate amylose content would be the best material for making porridge (4). Besides, when starch is used in biopolymer film production, the properties of biopolymer film is also influenced by above mentioned starch characteristics (5). Several indicators of starch characteristics are established, and these indicators are known as starch physicochemical and structural properties (4, 6).

A fundamental knowledge of the physicochemical and structural characteristics of a starch is necessary for further developing its utilization. Therefore, in this study, starch samples were extracted from the seeds of two *Cycas revoluta* grown in different environments. Then, the structural and physicochemical properties of these samples were systematically investigated. The objective of the current study is to reveal the characteristics of starch in seeds of *Cycas revoluta*, hence to provide useful information for later exploring its utilization in food and non-food industry.

## 2. Materials and methods

### 2.1. Materials

Two starch samples were used in this study. One (T_1_) was extracted from the seeds of *Cycad revoluta* grown in Shanghai City, China (121.4°E, 30.0°N); and the other (T_2_) was extracted from the seeds of *Cycad revoluta* grown in Guiyang City, Guizhou Province, China (106.7°E, 26.4°N).

### 2.2. Scanning electron microscopy (SEM)

The morphology of the starch granules was observed using an SEM (Scanning Electron Microscope VEGA3, TESCAN, Brno, Czech Republic).

### 2.3. Apparent amylose content (AAC)

Apparent amylose content was measured according to the method of Li et al. (4).

### 2.4. Particle size distribution

Particle size distribution of the starch granules was determined using a laser diffraction particle size analyzer (S3500, Microtrac, Montgomeryville, PA, United States).

### 2.5. Crystal properties

The diffraction pattern of starch samples was determined by an X-ray diffractometer (3 kW/*D8 ADVANCE Da Vinci, Bruker, Karlsruhe, Germany). An X-ray tube Cu-Kα (nickel filter) at 40 kV and 40 mA was used as the X-ray source. The diffraction angle (2θ) was 5°-40° with a step interval of 0.02°.

### 2.6. Pasting properties

The pasting properties were measured by a rapid visco analyzer (RVA4500, Perten Instruments, Hägersten, Sweden), and the data was recorded and processed by the Thermocline for Windows software. Sample preparation and measurement were based on the procedure described previously (6).

### 2.7. Textural properties

The starch gel formed after RVA test was further analyzed by a TA-XT2i Texture Analyzer (Stable Micro Systems, Godalming, United Kingdom) to determine the textural properties, following the previously published procedure (6).

### 2.8. Thermal properties

Thermal properties were measured by differential scanning calorimetry (Discovery DSC 25, TA Instruments, New Castle, DE, United States), based on the previously published procedure (6).

### 2.9. Fourier transform infrared (FTIR) spectrum analysis

Fourier transform infrared spectra was obtained via scanning starch samples using a Nicolet iZ-10 FTIR instrument (Thermo Fisher Scientific, Waltham, MA, United States). Starch sample (5 mg) was mixed with potassium bromide (250 mg) and pressed into a film-coated tablet. Wavenumbers from 400 to 4,000 cm^−1^ were measured at 4 cm^−1^ spectral resolution over 32 scans.

### 2.10. Branch chain-length distribution

High-performance anion-exchange chromatography (HPAEC) was performed to determine the branch chain-length distribution of amylopectin. Samples was analyzed by a CarboPac PA-100 anion-exchange column (4.0*250 mm; Dionex) using a pulsed amperometric detector (PAD; Dionex ICS 5000 system). Data were collected on the ICS5000 (Thermo Fisher Scientific, Waltham, MA, United States), and processed using chromeleon 7.2 CDS (Thermo Fisher Scientific, Waltham, MA, United States).

### 2.11. Molecular weight distribution analysis

Gel permeation chromatography-refractive index-multiangle laser light scattering detector (GPC-RI-MALLS) was used to measure the starch molecular weight. The differential refractive index detector (Optilab T-rEX, Wyatt Technology Co., Santa Barbara, CA, United States) was equipped with two tandem columns (300 × 8 mm, Shodex OH-pak SB-805 and 803; Showa Denko K.K., Tokyo, Japan). The data was recorded and processed using ASTRA6.1 software (Wyatt, Santa Barbara, CA, United States).

### 2.12. Average degree of branching

The Bruker BioSpin GmbH NMR spectrometer equipped with a tempering unit was used to measure the average degree of branching. Starch sample (10 mg) was mixed with 1 mL of deuterated dimethyl sulfoxide-d6 (DMSO-d6) and the solution was thoroughly mixed at 80°C overnight. The mixture was centrifuged at 12,000 rpm. Then, the supernatant was taken and transferred into an NMR tube. The ^1^H NMR scanning was performed 32 times, at a Larmor frequency of 500.23 MHz. Data were acquired and analyzed using MestReNova. Average DB was calculated with the following equation: DB (%) = (I-1,6) / (I-1,6 + I-1,4)*100, where I-1,4 is the integrated signal at 5.12 ppm and I-1,6 is the integrated signal at 4.77 ppm, respectively.

### 2.13. Statistical analysis

All traits were measured in duplicate. Data analysis was conducted with SPSS 25.0 statistical software program. Significance of differences between the means were determined by the independent t-test analysis (*p* < 0.05).

## 3. Results and discussion

### 3.1. Morphology of starch granules

**Figure 1:**
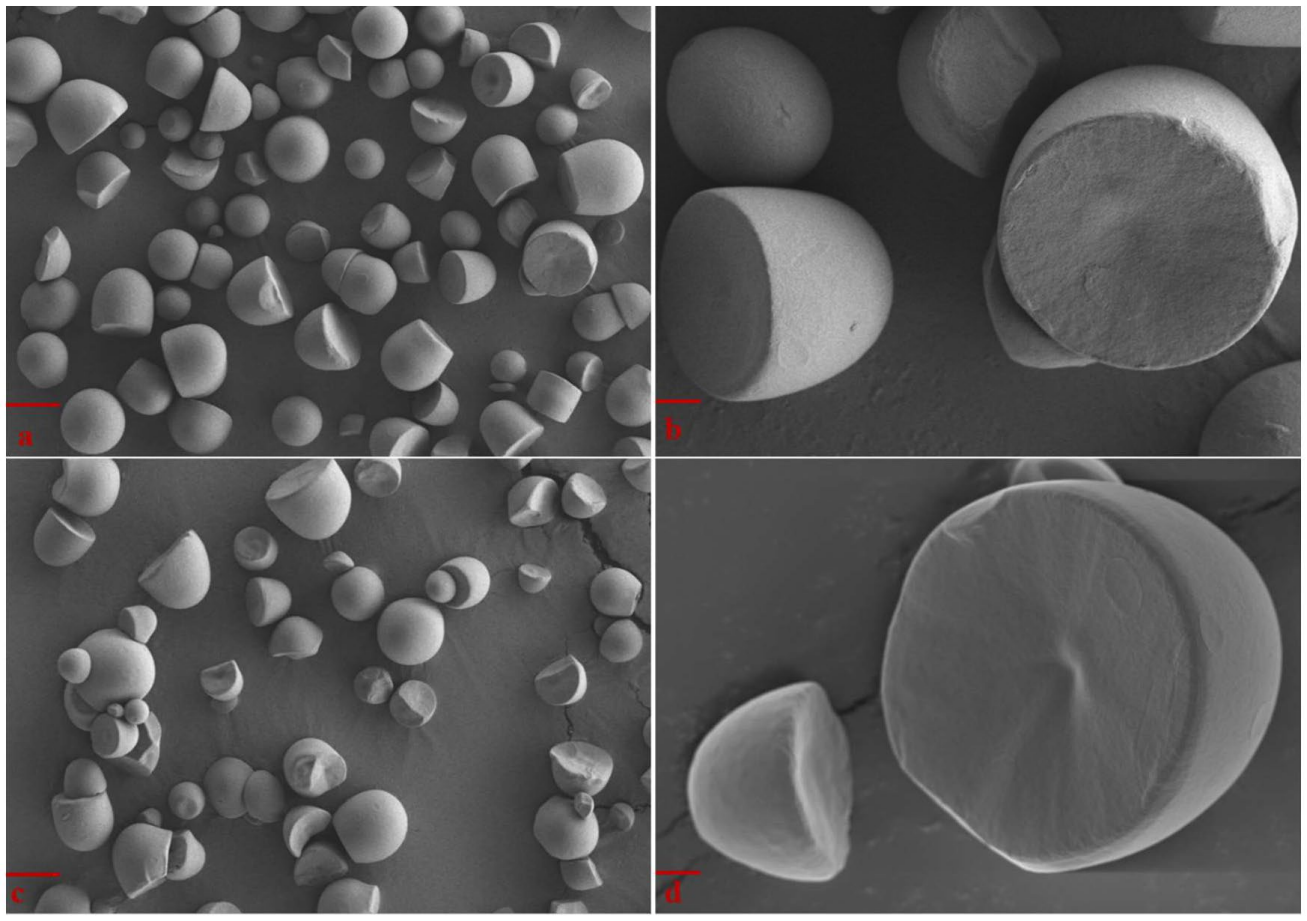
SEM images of *Cycad revoluta* starch granules **(A)** Sample T_1_, Bar = 10 μm; **(B)** Sample T_1_, Bar = 2 μm; **(C)** Sample T_2_, Bar = 10 μm; **(D)** Sample T_2_, Bar = 2 μm.

The morphology of *Cycad revoluta* starch granules observed by scanning electron microscope are shown in Figure 1. The *Cycad revoluta* starch granules showed unique morphology, they were spherical-truncated in shape with smooth surface, which is similar to the shape reported for *Lithocarpus dealbatus* and cassava (*Manihot esculenta*) starch (7–9). In contrast, rice and maize starch granules are polygonal, and potato starch granules are oval-shaped (10, 11).

### 3.2. Particle size distribution

Particle size distribution of *Cycad revoluta* starch samples were presented in Table 1. D (4,3) and D (3,2) represent volume and area diameters, respectively. D (0.1), D (0.5), and D (0.9) indicate 10, 50 and 90% of the starch granules were smaller than the values. The two *Cycad revoluta* starches were significantly different in D (3,2), D (4,3), D (0.9), D (0.5) (*p*<0.05), with mean values of these parameters of T_2_ were higher than those of T_1_. D (0.1) of T_1_ was higher than that of T_2_, but the difference was insignificant.

**Table 1:** Structural and physicochemical properties of *Cycad revoluta* starches.^a^

Property	Trait	Sample	
		T_1_	T_2_
Particle size distribution	D (3,2)/μm	12.8 ± 0.233^b^	14.5 ± 0.035^a^
	D (4,3)/μm	18.2 ± 0.905^b^	21.6 ± 3.82^a^
	D (0.1)/μm	7.86 ± 0.021^a^	7.79 ± 0.099^a^
	D (0.5)/μm	13.9 ± 0.354^b^	15.6 ± 0.191^a^
	D (0.9)/μm	26.0 ± 1.70^b^	27.3 ± 1.34^a^
Apparent amylose content	AAC	34.3 ± 0.948^a^	35.5 ± 0.792^a^
RVA	PV (cP)	5,700 ± 70.7^b^	5,927 ± 1.41^a^
	HPV (cP)	2,413 ± 21.9^b^	2,807 ± 9.19^a^
	CPV (cP)	4,364 ± 39.6^b^	4,688 ± 9.90^a^
	BD (cP)	3,288 ± 48.8^a^	3,121 ± 7.78^b^
	SB (cP)	−1,336 ± 31.1^a^	−1,239 ± 8.49^a^
	CS (cP)	1952 ± 17.7^a^	1882 ± 0.71^b^
Texture properties	HD (g)	77.0 ± 0.445^b^	106.9 ± 1.46^a^
	ADH (g.s)	−222.3 ± 3.81^b^	−173.1 ± 2.35^a^
	COH	0.469 ± 0.0^a^	0.427 ± 0.0^b^
DSC	T_o_ (°C)	73.7 ± 0.212^a^	69.8 ± 0.071^b^
	T_p_ (°C)	78.8 ± 0.354^a^	74.6 ± 0.0^b^
	T_c_ (°C)	85.6 ± 0.354^a^	81.2 ± 0.0^b^
	ΔH_g_ (J/g)	15.1 ± 0.566^a^	15.3 ± 0.707^a^
FTIR	1047/1022	1.89 ± 0.0^a^	1.73 ± 0.080^a^
	1022/995	0.379 ± 0.002^a^	0.414 ± 0.053^a^
Molecular weight distribution	Mn (kDa)	13,682 ± 904^a^	11,853 ± 3043^a^
	Mw (kDa)	62,213 ± 645^a^	52,240 ± 7099^a^
	Mw/Mn	4.56 ± 0.35^a^	4.48 ± 0.55^b^
	Rz	177 ± 5.52^a^	170 ± 1.27^a^
	Branching Degree (%)	4.85 ± 0.04^a^	3.83 ± 0.05^b^
GPC	Peak 1 (%)	46.5 ± 0.13^b^	53.8 ± 0.16^a^
	Peak 2 (%)	27.8 ± 0.15^a^	26.7 ± 0.04^b^
	Peak 3 (%)	25.7 ± 0.29^a^	19.5 ± 0.20^b^
HPEAC	DP ≤ 12	21.3 ± 0.071^b^	22.3 ± 0.283^a^
	DP 13–24	47.9 ± 0.071^a^	48.0 ± 0.212^a^
	DP 25–36	13.6 ± 0.141^a^	13.6 ± 0.071^a^
	DP ≥ 37	17.3 ± 0.0^a^	16.2 ± 0.424^a^

^a^All the values are means ± standard deviations. Different letters in the same column indicate significant difference (*p* < 0.05).

AAC, apparent amylose content; ADH, adhesiveness; BD, break down; COH, cohesiveness; CPV, cold paste viscosity; CS, consistency; DP, degree of polymerization; DSC, differential scanning calorimetry; FTIR, fourier transform infrared spectrometer; GPC, gel performance chromatography; GT, gelatinization temperature; HD, hardness; HPEAC, high-performance anion-exchange chromatography; HPV, hot paste viscosity; Mn, number-average molecular weight; Mw, weight-average molecular weight; Ptime, peak time; PT, pasting temperature; PV, peak viscosity; RVA, rapid visco-analyzer; Rz, z-radius of gyration; SB, setback; To, onset temperature; Tc, conclusion temperature; Tp, peak temperature; XRD, X-ray diffraction; ΔHg, enthalpy of gelatinization.

The *Cycad revoluta* starch granules had an average area diameter smaller than 15 μm, and volume diameter less than 20 μm. The T_1_ and T_2_ samples had mean area diameter (D (3,2)) of 12.8 μm and 14.5 μm, respectively. D (0.9) were 26.0 μm and 27.3 μm for T_1_ and T_2_, respectively. Generally, judging on the value of mean area diameter, the *Cycad revoluta* starch granules sized similarly to maize, cassava, and sweet potato starch, and smaller than potato starch, but larger than rice starch (8, 12–16).

### 3.3. Apparent amylose content

The two *Cycad revoluta* starches had significantly different apparent amylose content. AAC was 34.3% for T_1_ sample while 35.5% for T_2_ sample (Table 1). In rice, AAC could be divided into five groups: waxy (0–2%), very low (5–12%), low (12–20%), intermediate (20–25%) and high (25–33%) (17). As reported previously, AAC ranged from 18.3 to 25.3% in 34 foxtail millet genotypes (4), 2.09 to 35.25% in 192 maize landraces (18), and from 18.9 to 29.4% in 29 potato accessions (19). Therefore, the *Cycad revoluta* starch had much higher AAC than most commonly consumed starches.

### 3.4. Pasting properties

All parameters of pasting properties determined by RVA are presented in Table 1. T_1_ and T_2_ varied significantly in all parameters except SB (Table 1). The sample (T_2_) with higher AAC also had higher PV, HPV, CPV. According to previous reports, AAC do have significantly positive correlation to HPV and CPV in some cases [(19–21)], however, AAC usually have negative rather than positive correlation to PV (19, 20, 22). Nevertheless, PV is not only affected by amylose content, but also determined by amylopectin structure. Singh et al. (23) reported that the greater DP of amylopectin chains led to higher values of viscosity. In this respect, the differences on viscosity values between the two *Cycad revoluta* starch samples could attribute to a complex variation in starch characteristics.

PV, HPV, CPV, BD, SB, and CS were 5,700 and 5,927 cP, 2,413 and 2,807 cP, 4,364 and 4,688 cP, 3,288 and 3,121 cP, −1,336 and − 1,239 cP, 1952 and 1882 cP for the two samples. Since viscosity values can be substantially affected by starch concentration, we compared our result specifically to those using same starch concentration in RVA test. The viscosity values of *Cycad revoluta* starches were within the range of rice (6), and were higher than that of most of cassava genotypes (24). The viscosity values of *Cycad revoluta* starches should be lower than that of most potato cultivars, as the latter ones exhibited similar or higher viscosity values, even under a lower starch concentration (19).

### 3.5. Gel textural properties

Gel textural properties are summarized in Table 1. HD, ADH and COH of the two samples were 77.0 and 106.9 g, −222.3 to −173.1 g.s, 0.469 to 0.427, respectively (Table 1). All the three parameters varied significantly between the two samples, and *Cycad revoluta* starches can form harder gels than rice starch, under same concentration. All of this could be due to the variance in starch composition. As suggested by previous reports, amylose content is positively correlated to gel hardness and stickiness, and the structure of amylopectin also affects the texture of starch gel (25–27).

### 3.6. Thermal properties

**Figure 2:**
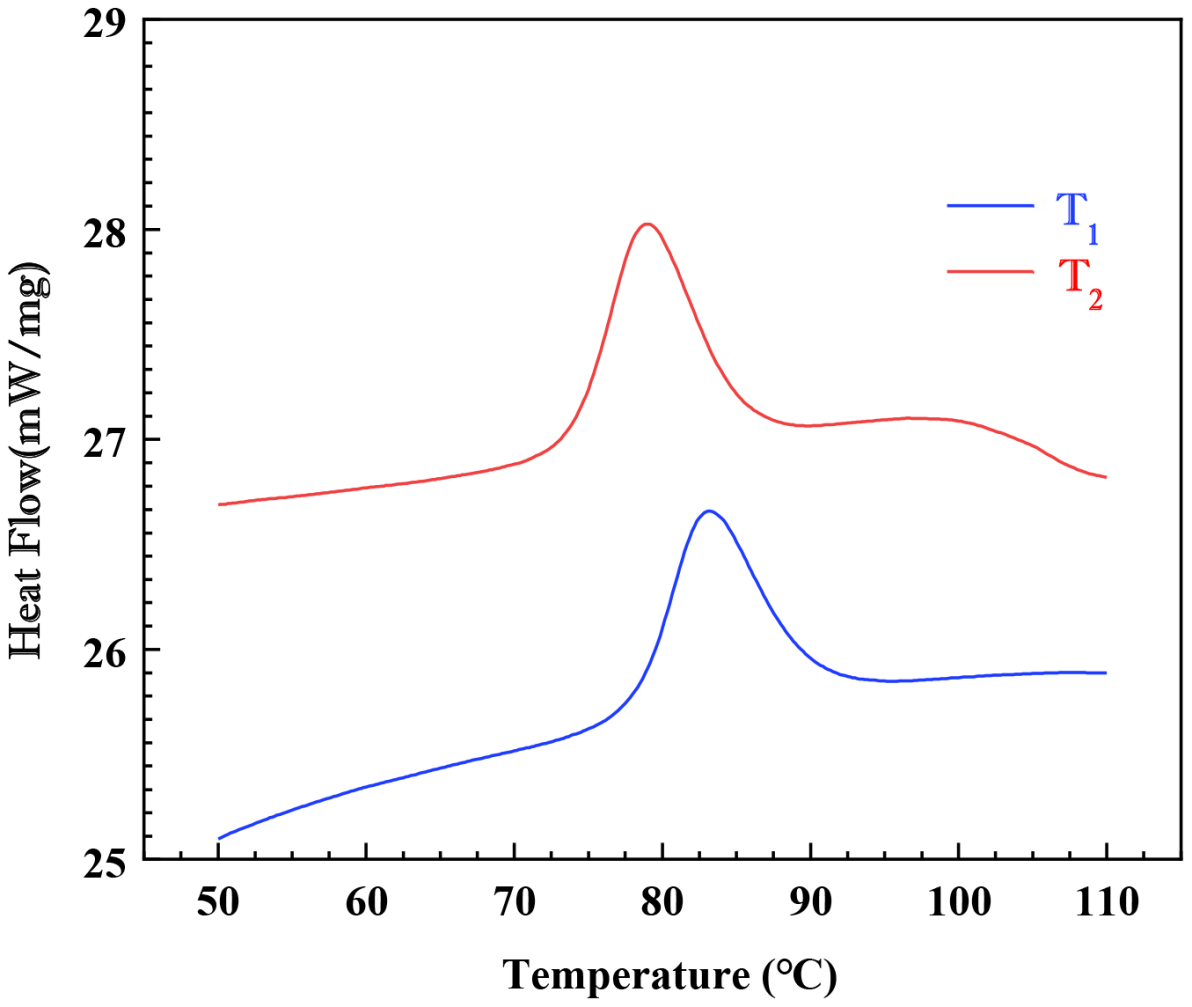
DSC profiles of the two *Cycad revoluta* starches.

Gelatinization temperatures and transition enthalpies of *Cycad revoluta* starches are presented in Table 1 and DSC profiles are shown in Figure 2. A single endothermic conversion was observed in the DSC profile for each sample (Figure 2). Onset (T_o_), peak (T_p_), conclusion (T_c_) and gelatinization enthalpy (∆H_g_) were 73.7 and 69.8°C, 78.8 and 74.6°C, 85.6 and 81.2°C, and 15.1 and 15.3 J/g, respectively. The two samples varied significantly (*p*<0.05) in all thermal parameters except ΔH_g_.

The values of thermal properties of *Cycad revoluta* starches are within the range of that reported for 163 rice accessions, wherein T_o_, T_p_, and T_c_ ranging from 59.2 to 76.6°C, 66.7 to 81°C, and 71.8 to 87.8°C, respectively (6). In seven maize samples, gelatinization temperature (T_p_) varied from 72.5 to 75.7°C (28). Ahmed et al. (19) reported a 66.1°C-71.1°C range of T_p_ variation in 29 potato genotypes. To conclude, the gelatinization temperatures of *Cycad revoluta* starches are similar to that of commonly consumed cereal starchers, but higher than potato starch.

### 3.7. Crystallinity

There are three main types of crystallinity in starch based on XRD patterns: A-type starch has peaks at about 15°, 17°, 18°and 23°, and usually exists in cereal starch. B-type having peaks at approximately 5°, 6°, 15°, 17°, 18° and 23° are usually found in tuber starch. C-type is a combination of A-and B-type, and is usually found in bean starches (29, 30).

**Figure 3:**
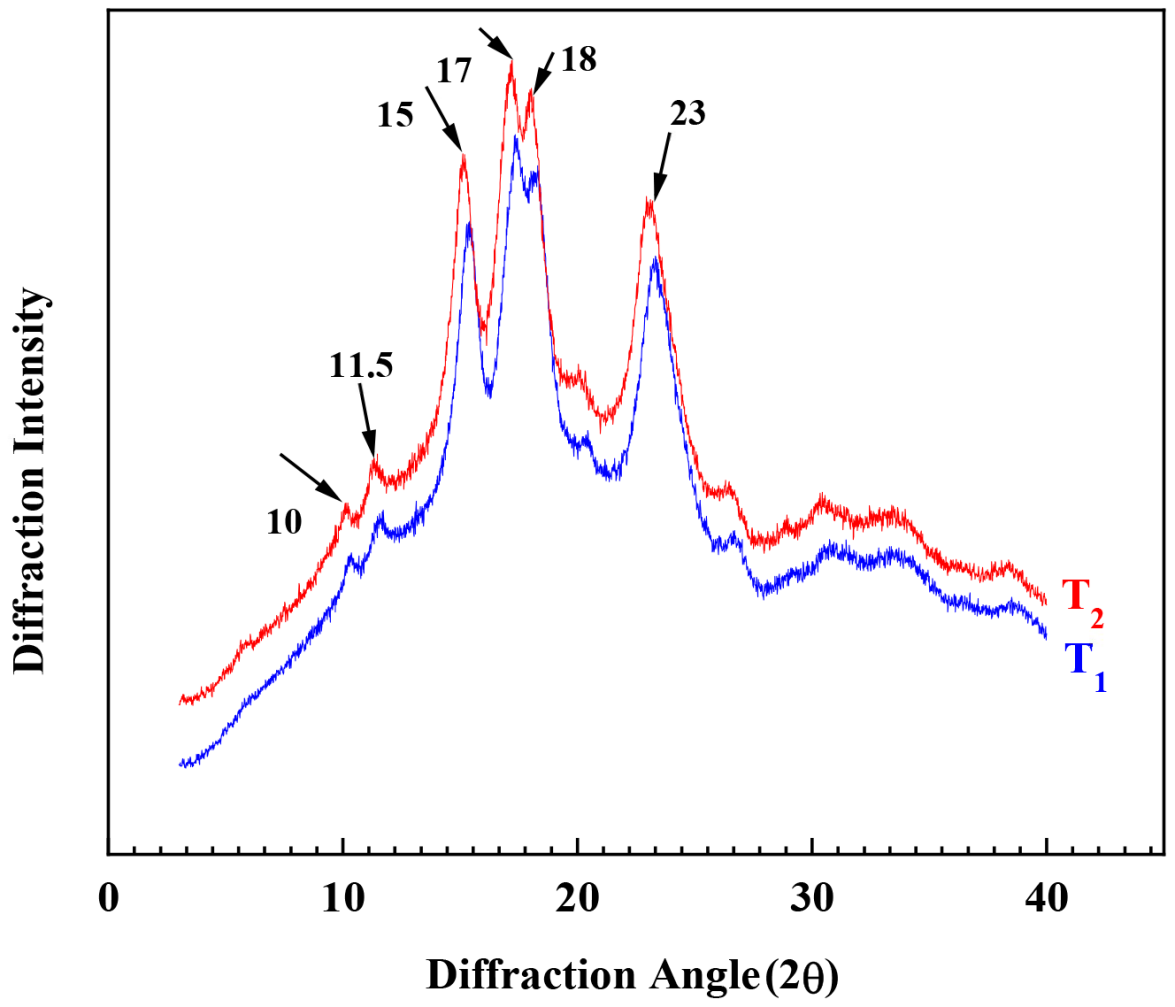
XRD patterns of the *Cycad revoluta* starches.

XRD patterns of *Cycad revoluta* starches were shown in Figure 3. Peaks of moderate intensity were observed at 10°and 11.5°, followed by a single peak with high intensity at 15°, an unresolved double peak at 17° and 18°, and a broad peak at 23°. This XRD pattern is previously reported in maize and cassava starches and was regarded as A-type crystalline structure (Poto et al., 2018; (31)).

### 3.8. FT-IR analysis

The FT-IR was conducted to determine the short range order of the *Cycad revoluta* starches. The crystalline region, amorphous region and hydrated carbohydrate helices of the starch molecule are associated to the absorption peaks at 1047 cm^−1^, 1,022 cm^−1^ and 995 cm^−1^, respectively. Therefore, 1,047/1022 ratio is an intensity indicator of crystalline and amorphous regions while 1022/995 ratio indicates the formation of double helix of starch molecules (32). As presented in Table 1, the two samples had similar absorption peaks, and did not differ significantly in neither of 1047/1022 and 1022/995. However, the 1047/1022 ratio of *Cycad revoluta* was substantially higher than that of cereal and potato starches (33, 34), suggesting a clear difference in starch structure between *Cycad revoluta* and traditional common starches.

### 3.9. Molecular weight distribution

The z-radius of gyration (Rz), number-average molecular weight (Mn), weight-average molecular weight (Mw) and the degree of the dispersion of the molecular weight distribution (Mw/Mn) of *Cycad revoluta* starches are summarized in Table 1. Rz, Mn, Mw, Mw/Mn in each sample were 177 nm, 13,682 KDa, 62,213 KDa, 4.56 for T_1_, and 170 nm, 11,852 KDa, 52,240 KDa, 4.48 for T_2_. The two samples only differed significantly in Mw/Mn (*p*<0.05).

The high Mw is an indicator for highly polymerized amylopectin, and the high ratio of Mw/Mn indicates that the molecular weight distribution of starch is highly dispersed (35). Higher Rz value indicates higher branching degree, as Rz means the theoretical probability of finding a molecule at a given distance from the center (36). Compared to conventional main-stream starches, *Cycad revoluta* starches showed similar Mw, but smaller Mn and higher Rz values than rice starches (37), and much higher Mw and Mn than both maize and cassava starch (38, 39). The branching degree of T_1_ was significantly higher than that of T_2_ (*p*<0.05), this is in consistent with the variation pattern in Mw, Mn, Mw/Mn, and Rz between the two samples (Table 1).

### 3.10. Relative molecular weight distribution

**Figure 4:**
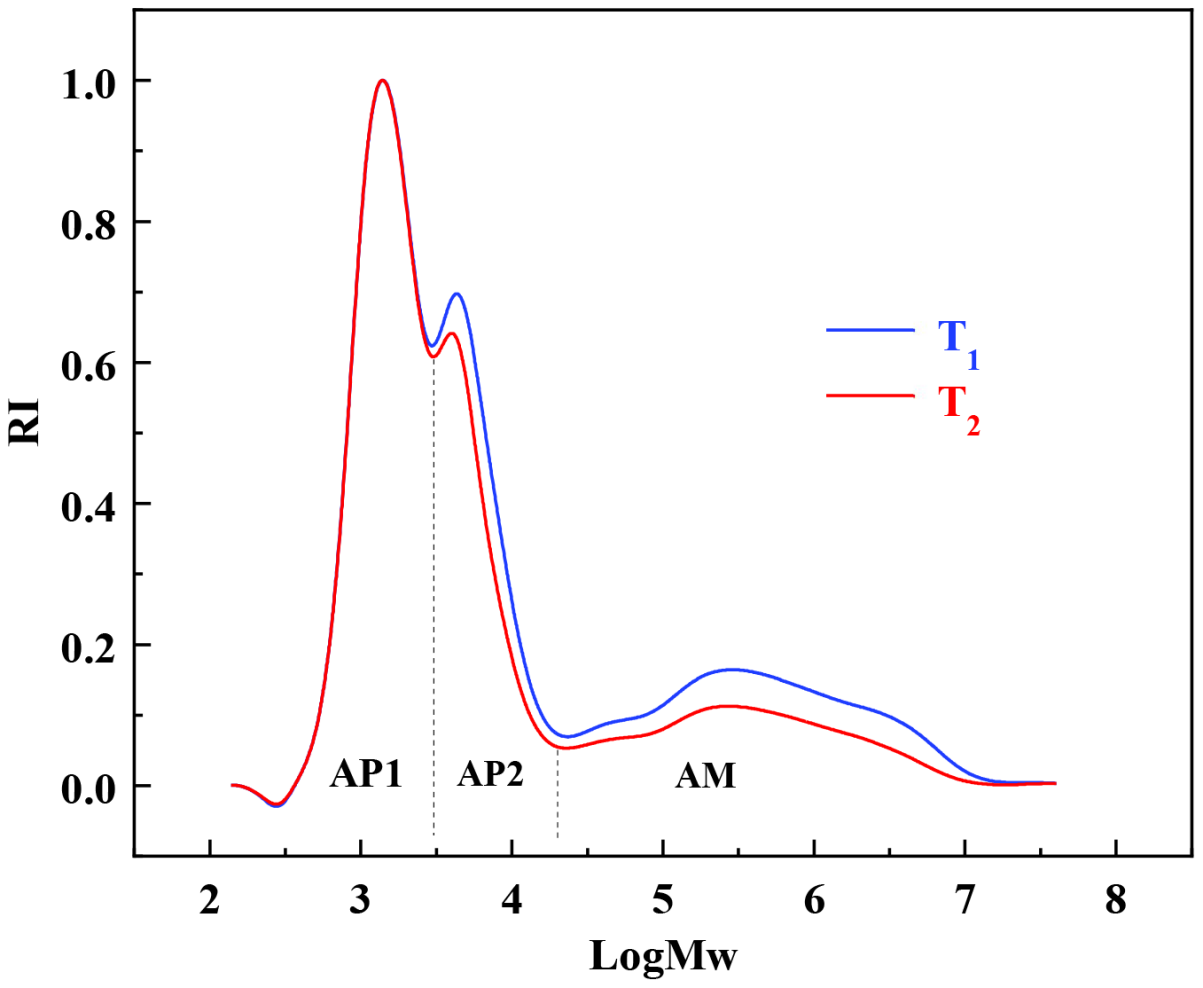
The relative molecular weight distributions of the *Cycad revoluta* starches.

Based on the relative molecular weight distribution analysis, conducted by gel performance chromatography (GPC), branching patterns of the isoamylase-debranched *Cycad revoluta* starches were determined. The results are shown in Figure 4 and Table 1. The three peaks in Figure 4 represented amylopectin with short-branch chains (AP1), amylopectin with long-branch chains (AP2) and amylose molecules (AM), respectively (40).

The ratios of the three peaks were 46.5 and 53.8%, 27.8 and 26.7%, 25.7 and 19.5%, respectively, in the two *Cycad revoluta* starch samples. T_2_ had more proportions of short-branch chains (53.8% *VS* 46.5%) but less long-branch chains (27.8% *VS* 26.7%) and amylose chains (19.5% *VS* 25.7%) than T_1_ (*p*<0.05). Peng et al. (41) concluded in rice study that higher proportion of amylopectin short chains associated with higher peak viscosity (PV) and breakdown value (BD), and a softer and stickier texture (HD). This is consistent with the data of the current study. As shown in Table 1, the *Cycad revoluta* starch sample with higher proportion of amylopectin short chains (T_2_) did have higher PV, BD and lower HD values than that of sample with lower proportion of amylopectin short chains (T_1_).

### 3.11. Chain length distribution of the debranched amylopectin

**Figure 5:**
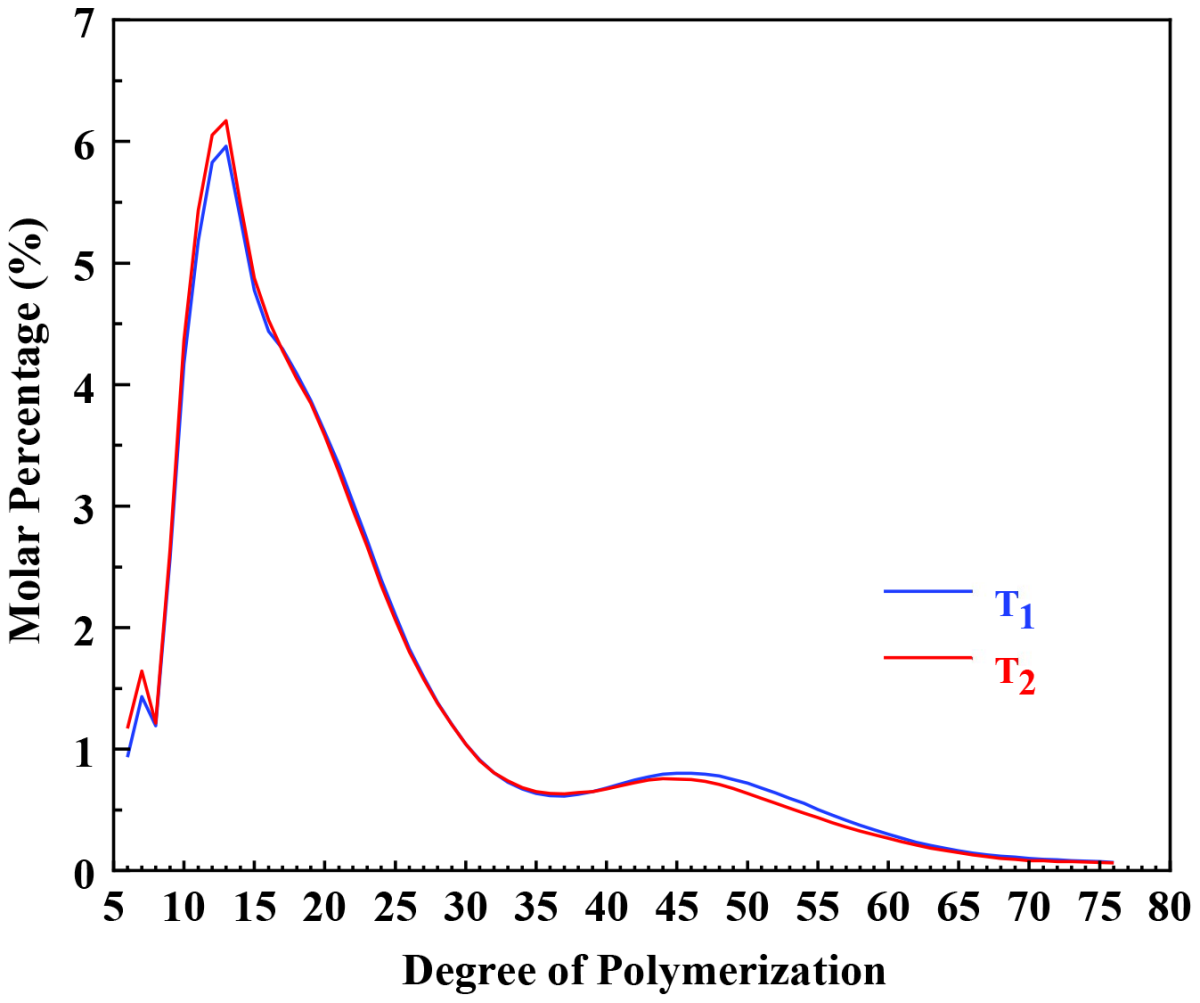
The chain length distribution of debranched amylopectin of the *Cycad revoluta* starches.

High-performance anion-exchange chromatography-pulsed amperometric detection (HPAEC-PAD) was used to determine the chain length distribution of amylopectin in this study. The results are shown in Figure 5 and Table 1. According to the degree of polymerization (DP) and the model of amylopectin cluster, branched chains can be grouped into four classes: A (DP 6–12), B1 (DP 13–24), B2 (DP 25–36), and B3 (DP ≥ 37) (42, 43).

As presented in Table 1, the proportions of A, B1, B2, and B3 chains of the two samples were 21.3, 47.9, 13.6, 17.3 and 22.3%, 48.0, 13.6, 16.2%, respectively. The two *Cycad revoluta* starches only varied significantly in proportion of A chain.

Compared to cereal starches, the patterns of amylopectin chain distribution of *Cycad revoluta* starches were very different from that of wheat and corn, but similar to some rice varieties (44–46). Generally, the *Cycad revoluta* amylopectin showed much higher proportion of long chains (B2 + B3 chains), but much lower proportion of short chains (A chain) than that of wheat starch (46), and contained much higher proportion of A and B1 chains, but much lower proportion of B2 and B3 chains than that of corn starch (44). Compared to potato starch, *Cycad revoluta* starch had more B1 chains, but less B2 and B3 chains, and similar proportion of A chain (47).

## 4. Conclusion

The structural and physicochemical properties of two *Cycad revoluta* starch samples were systematically investigated in this study. The *Cycad revoluta* starch granules were spherical-truncated in shape with smooth surface, and had a small size (mean area diameter < 15 μm). XRD analysis revealed that the *Cycad revoluta* starches had A-type crystallinity. During gelatinization, the *Cycad revoluta* starches showed similar viscosity profile with that of some rice and potato varieties, but higher gelatinization temperature than potato starch. The branch chain length distribution analysis revealed that the *Cycad revoluta* starches were structurally similar to some rice varieties, but totally different from that of corn, wheat and potato starches. In general, the *Cycad revoluta* starches had distinctive characteristics and its application remained explored in later studies, and the current study provides fundamental information for application of *Cycad revoluta* starches in food and non-food industries.

## Data availability statement

The raw data supporting the conclusions of this article will be made available by the authors, without undue reservation.

## Author contributions

KL: conceptualization, resources, supervision, data curation, writing-original draft, and writing-review & editing. TZ: data curation, methodology, and investigation. HR, WZ, and SH: software. YG: methodology. HC: writing-review & editing and funding acquisition. All authors contributed to the article and approved the submitted version.

## Funding

The authors would like to thank Shantou Science and Technology Bureau (grant no. STKJ2021024), Guizhou University Natural Science Project (2020-23), Guizhou University Seed Program (2020-26), and The Opening Foundation of National Laboratory of Hazard Factors and Risk Prevention of Agricultural Product Quality and Safety (2021DG700024-KF202209) for financial support.

## Conflict of interest

The authors declare that the research was conducted in the absence of any commercial or financial relationships that could be construed as a potential conflict of interest.

## Publisher’s note

All claims expressed in this article are solely those of the authors and do not necessarily represent those of their affiliated organizations, or those of the publisher, the editors and the reviewers. Any product that may be evaluated in this article, or claim that may be made by its manufacturer, is not guaranteed or endorsed by the publisher.
